# The role of quantitative mass spectrometry in the discovery of pancreatic cancer biomarkers for translational science

**DOI:** 10.1186/1479-5876-12-87

**Published:** 2014-04-05

**Authors:** Daniel Ansari, Linus Aronsson, Agata Sasor, Charlotte Welinder, Melinda Rezeli, György Marko-Varga, Roland Andersson

**Affiliations:** 1 Department of Surgery, Clinical Sciences Lund, Lund University, and Skåne University Hospital, SE-221 85 Lund, Sweden; 2 Department of Pathology, Clinical Sciences Lund, Lund University, and Skåne University Hospital, Lund, Sweden; 3 Department of Oncology, Clinical Sciences Lund, Lund University, Lund, Sweden; 4 Clinical Protein Science & Imaging, Biomedical Center, Department of Measurement Technology and Industrial Electrical Engineering, Lund University, Lund, Sweden

**Keywords:** Biomarker, Mass spectrometry, Diagnostics, Pancreatic cancer, Proteomics

## Abstract

In the post-genomic era, it has become evident that genetic changes alone are not sufficient to understand most disease processes including pancreatic cancer. Genome sequencing has revealed a complex set of genetic alterations in pancreatic cancer such as point mutations, chromosomal losses, gene amplifications and telomere shortening that drive cancerous growth through specific signaling pathways. Proteome-based approaches are important complements to genomic data and provide crucial information of the target driver molecules and their post-translational modifications. By applying quantitative mass spectrometry, this is an alternative way to identify biomarkers for early diagnosis and personalized medicine. We review the current quantitative mass spectrometric technologies and analyses that have been developed and applied in the last decade in the context of pancreatic cancer. Examples of candidate biomarkers that have been identified from these pancreas studies include among others, asporin, CD9, CXC chemokine ligand 7, fibronectin 1, galectin-1, gelsolin, intercellular adhesion molecule 1, insulin-like growth factor binding protein 2, metalloproteinase inhibitor 1, stromal cell derived factor 4, and transforming growth factor beta-induced protein. Many of these proteins are involved in various steps in pancreatic tumor progression including cell proliferation, adhesion, migration, invasion, metastasis, immune response and angiogenesis. These new protein candidates may provide essential information for the development of protein diagnostics and targeted therapies. We further argue that new strategies must be advanced and established for the integration of proteomic, transcriptomic and genomic data, in order to enhance biomarker translation. Large scale studies with *meta data* processing will pave the way for novel and unexpected correlations within pancreatic cancer, that will benefit the patient, with targeted treatment.

## Introduction

Increasing demands in health care today pose high expectations and directives for the research community to develop solutions that can improve clinical outcome with improved cost efficiency. The development of new diagnostic biomarkers has a great potential and solutions are being tested both in the pharmaceutical industry and within academic medicine settings [1-4]. One such area of unmet need is in the diagnosis and treatment of pancreatic cancer.

Pancreatic cancer is the 10th most common cancer in the Western world [5]. With an overall 5-year survival rate less than 5%, it has the lowest survival rate among human cancers and has become the 4th leading cause of cancer-related death [6]. An estimated 277,000 new cases of pancreatic cancer are diagnosed globally each year with approximately 266,000 deaths [7]. The total health-care costs and loss of productivity related to pancreatic cancer are high, and increasing. In Sweden (population 9.5 million), the yearly economic costs for pancreatic cancer was estimated between EUR 86 and 93 million [8]. Pancreatic cancer has a low survival as symptoms often are vague and today there are no established markers for screening, and early diagnosis. Approximately 85-90% of all patients with pancreatic cancer are diagnosed with advanced and inoperable disease. It has been shown that an improved survival is achievable when tumors are detected at an early stage. For example, 5-year survival rates of 50% have been reported in tumors <2 cm [9] while for tumors <1 cm the reported 5-year survival rate was found to be close to 100% [10].

For pancreatic cancer, the only biomarker approved by the FDA and clinically used is carbohydrate antigen 19–9 (CA 19–9). It has been used since the 1980s as a marker for recurrence and progression, based on a study where CA 19-9 outperformed carcinogenic antigen (CEA) in predicting recurrence following surgery [11]. It has been found that CA 19–9 has a relatively high sensitivity and specificity (about 80% as seen in 22 studies) [12] which is superior to other markers, including CEA, CA-50 and DUPAN-2 [12-14]. However, the low positive predictive value taken together with the fact that benign causes and all forms of biliary obstruction, can trigger an increase in CA 19–9 serum levels, diminish its utility as a screening tool [12,15]. Additionally, 10% of the population do not have the enzyme activity genotype (le/le) and consequently cannot synthesize CA 19–9 [16]. Despite this, CA 19–9 is considered the best serum marker for pancreatic cancer and the one against which new markers should be judged [17].

Systems biology and one of its supporting disciplines, proteomics, has evolved widely over the past decade, providing novel methods to tackle increasing challenges in health care. With increasing knowledge and improved techniques, the field of proteomics has introduced a new paradigm for detecting disease at an early curable stage. Proteomics is no longer a protein expression methodology for specialists. On the contrary, proteomics is used and applied to all areas of life science today. Proteomics is especially applicable in the treatment of pancreatic cancer, where successful treatment is directly related to early intervention [18]. Today the accuracy, sensitivity and specificity of proteomics is directly linked to the development of high performance mass spectrometry instruments for the measurement of analytes in clinical samples. Modern high-end mass spectrometry platforms enable high-resolution power in sequencing, as well as identifying post-translational-modifications [19]. Mass spectrometry also offers a way to localize new disease regulating proteins for target-specific anti-cancer drugs, renewing a hope for a breakthrough in the treatment outcome of pancreatic cancer, like the one seen in other malignancies, with personalized medicines such as Imatinib in Philadelphia chromosome-positive acute myeloid leukemia (Ph+AML) [20] and Gefitinib (IRESSA), as well as Tarceva (Erlotinib) in the therapy of non-small cell lung cancer [2].

The basic concept that health and disease differ in gene and protein regulatory networks is well established. Genome sequencing has revealed a complex set of genetic alterations in pancreatic cancer such as point mutations, chromosomal losses, gene amplifications and telomere shortening that drive cancerous growth through specific signaling pathways [21,22]. Functional studies are needed in order to investigate which pathways that are critical on a protein level. Today’s mass spectrometry instrumentation and its merits were pioneered by Fenn’s group on electrospray ionization [23], while Karas and Hillenkamp [24] and Tanaka [25] developed Matrix Assisted Laser Desorption Ionization – “MALDI” mass spectrometry. These were milestone inventions that revolutionized modern Post-Genomic research, and were awarded the Nobel Prize in 2002. These inventions have further changed our way of working with clinical protein science today.

Increasing our understanding of the biological drivers of pancreatic cancer could serve to identify new diagnostic and therapeutic biomarkers. Here, we give an overview on the role of quantitative mass spectrometry in the discovery, validation and translation of new biomarkers for pancreatic cancer patients. We argue for a global, integrated proteomic and transcriptomic work flow in the analysis of pancreatic tumors. The establishment of custom databases and comparison with genomic data such as ENCODE, is mandatory in order to discover and utilize new molecular entities within disease mechanisms.

## The biomarker concept

The biomarker concept is not a new invention *per se*. Its utilization has been exploited in both medicine, as well as within the drug development process. However, a distinction should be made to the surrogate marker, that is a definite indicator of a medical, or pathophysiological event. The National Institutes of Health (NIH) Biomarker working group defined a biomarker as *“a characteristic that is objectively measured and evaluated as an indicator of normal biologic processes, pathogenic processes, or pharmacologic response to a therapeutic intervention*[26]*.”*

Biomarkers can be further divided with respect to their utility in care [27]:

**Diagnostic biomarkers:** Detect onset, recurrence after surgery, supplying therapy guidance, or identifying progression of disease.

**Prognostic biomarkers:** State the natural disease process and can assist in treatment planning. Give insight about survival and recurrence patterns.

**Predictive biomarkers:** Predict the response to treatment.

Considering the disposition in clinical studies, biomarkers can be divided into several classes [2]:

1. Primary biomarkers (low abundant), such as receptor signaling kinases.

2. Secondary biomarkers (low/medium abundant), indirect biomarkers that are a resulting outcome of the signaling pathway biology.

3. Tertiary biomarkers (medium/high abundant), are proteins that are associated with functional changes in disease.

4. Disease biomarkers (can be low/medium/high abundant).

Proteomic profiling in the search for pancreatic cancer biomarkers is usually based on tissue, pancreatic juice (the secretions of the exocrine portion of the pancreas), and/or blood (serum or plasma).

As there are a number of disease mechanisms involved in pancreatic cancer, we are not at a stage where we have a disease-linked targeted route that provides efficacious outcomes. That is a challenge in itself. This is one of the salient reasons for using tissue samples that represent large disease regions in the pancreas in the hunt for disease related biomarkers. As such, by studying the tissue compartment where the disease occurs, we gain in the understanding of the origin and the basis of disease mechanisms, and protein drivers involved in that origin, as well as progression of disease. Once we have built an understanding of the changes in protein expression in the diseased tissue, we are facilitated to test for similar markers in other body fluids such as blood. When analyzing tissue in proteomic studies, formalin-fixed, paraffin-embedded (FFPE) tissue is more accessible than snap-frozen tissue specimens. FFPE tissue specimens are clinically and pathologically well defined, which allow a direct correlation of histological observations with proteomic analysis [28,29]. Recent developments in methodologies of protein extraction techniques from FFPE samples provide new opportunities for larger sets of study materials to be used in the survey of protein expression. The utility of laser microdissection along with FFPE, followed by proteomic studies, may allow the isolation of cancer cells and stromal cells to accurately determine the proteome profile of different tumor compartments [30,31].

Pancreatic juice is a biofluid which is in contact with proteins directly secreted from the pancreatic ducts. The possibility of shed cancer cells, makes the juice a rich biomarker source, and an opportunity due to the cancer-specific proteins expressed in these samples.

The human plasma proteome is the most accessible biofluid, and has the potential to significantly improve disease diagnosis and therapeutic monitoring [32]. However, given the low abundance in serum and plasma of known cancer biomarkers [33], new proteomic technologies are constantly being developed and refined to provide sufficient depth of analysis for biomarker quantification.

## Modern high-end mass spectrometry

Traditionally, two-dimensional gel electrophoresis (2-DE), resolving the proteome at a protein level, has in the past been extensively used in proteomic studies. The method utilizes the net charge of proteins at different pH values (isoelectric focusing) and the molecular weight of proteins to separate them in a two-dimensional pattern in the gel medium. The protein “spots” generated can then be stained (fluorescent tags), and consequently analyzed with different methods to identify and quantify the specific proteins. 2-DE was first introduced in 1975 by O’Farell [34] and gel electrophoresis has since experienced great improvements also within the context of pancreatic cancer [35,36]. However, it still carries limitations in gel reproducibility, and has a poor representation of highly basic/acidic, low abundant proteins and membrane proteins. A major limitation with 2-DE, is the inherent difficulty, to identify the proteins by MS-sequencing, as there is a limitation in excising proteins from the polymeric matrix of the gels. In addition, the low accuracy when comparing multiple proteins and difficulties in automation render this method unreliable and it may need to stand aside for the new gel-free mass spectrometric techniques [37]. In clinical protein science, new and future complementary technologies, like quantitative mass spectrometry including protein shotgun sequencing has been instrumental in these achievements [38-40]. One approach that has gain most acceptance over the last decade is liquid phase separation interfaced on-line with mass spectrometry [2,41,42]. It can be used for the identification and quantification of proteins across multiple sample sets. It can be used for protein deep mining studies (biomarker discovery) and targeted protein quantification (biomarker validation). It can also be used to detect post-translational modifications (PTMs).

## Protein deep mining

Protein deep mining studies can be performed using either label-free or labeled mass spectrometric approaches. These approaches have recently gained interest in the context of pancreatic cancer. Examples of candidate proteins have been identified. All of these biomarker candidates have been validated in tissue or biofluids (pancreatic juice, serum or plasma) of pancreatic cancer patients (as shown in Table 1).

**Table 1:** Protein deep mining studies in pancreatic cancer using mass spectrometry

**Technique**	**Sample type**	**Groups**	**Discovery phase**	**Biomarker candidates**	**References**
Label-free	Tissue	PC, N	1,009 proteins in total; 422 upregulated proteins in PC	ASPN, LTBP2, TGFBI	Turtoi et al. [49]
	Tissue	PC, N	1,229 proteins in total; 499 upregulated in PC	ECH1, GLUT1 (GTR1), OLFM4, STML2	Takadate et al. [30]
	Tissue	PC, CP, N	525 proteins in total; 21 upregulated proteins in PC	ANXA4, FN1	Paulo et al. [65]
	Plasma	PC, N	53,009 MS peaks	CXCL7	Matsubara et al. [69]
SILAC	Cell lines	PC, N	195 proteins in total; 68 upregulated proteins in PC; 5 biomarker candidates validated in pancreatic cancer tissue	CD9, HSPG2, APOE, SDF4, ITGB1	Grønborg et al. [81]
	Serum	PC, N	1,065 proteins in total; 121 upregulated proteins in PC	BCAM, ICAM1	Yu et al. [ 94]
ICAT	Tissue	PC, N	656 proteins in total; 151 upregulated proteins in PC	ANXA2, ITGB1	Chen et al. [100]
	Pancreatic juice	PC, N	105 proteins in total; 30 upregulated proteins in PC	IGFBP2	Chen et al. [103]
Acrylamide- labeling	Plasma	PC, CP, N	1,340 proteins in total; 95 and 87 proteins with ≥1.5 fold difference in PC compared to N and CP, respectively	ICAM1, TIMP1	Pan et al. [106]
TMT	Serum	PC, N	752 proteins in total	APOA4, F12, GSN, LTF	Sinclair et al. [110]
ICAT, iTRAQ	Tissue	PanIN-3, PC, CP, N	770 proteins in total; 70 proteins upregulated and 133 downregulated in PanIN-3	ACTN4, LAMB1, LGALS1	Pan et al. [119]

ACTN4, actinin alpha-4; ANXA2, annexin A2; ANXA4, annexin A4; APOE, apolipoprotein E; APOA4, apolipoprotein A-IV; ASPN, asporin; BCAM, basal cell adhesion molecule; CP, chronic pancreatitis; CXCL7, CXC chemokine ligand 7; ECH1, enoyl CoA hydratase 1; F12, coagulation factor XII; FN1, fibronectin 1; GLUT1 (GTR1), glucose transporter member 1; GSN, gelsolin; HSPG2, heparan sulfate proteoglycan 2 (perlecan); ICAM1, intercellular adhesion molecule 1; IGFB2, insulin-like growth factor-binding protein 2; ITGB1, integrin beta-1; LAMB1, laminin beta-1; LGALS1, galectin-1; LTBP2, latent transforming growth factor beta binding 2; LTF, lactotransferrin; N, normal; OLFM4, olfactomedin-4; PanIN-3, pancreatic intraepithelial neoplasia; PC, pancreatic cancer; SDF4, stromal cell derived factor 4; STML2, stomatin-like protein 2; TGFBI, transforming growth factor beta-induced; TIMP1, metalloproteinase inhibitor 1.

### Label-free quantification

Label-free quantification is based on the signal intensity created by the peptides in the mass spectrometer. The quantification is often performed using peak-intensity [43,44] or spectral counting [45-47]. These are straightforward techniques compared to labeled procedures. First, the individual samples are processed and analyzed by LC-MS. The LC separation step is used to reduce the complexity of analytes in the sample. Over 20,000 circulating peptides have been identified in human plasma [48]. Normalization is required to minimize the affection of slightly different constitutions of samples. Several peptides per protein should be quantified to certify correct annotations. To detect low-abundant proteins, there is a need for high-resolution multidimensional chromatography. The orthogonal approach is crucial in order to utilize additive parts within the protein structure that aids in the chromatographic separation mechanisms [2].

Recently in a study, Turtoi et al. [49] used label-free quantification of pancreatic cancer and normal pancreas tissues to identify systemically accessible proteins, defined as proteins located on the outer surface of the cell membrane and/or in the extracellular matrix, having the potential to serve as targets for diagnostic and therapeutic approaches. Eleven selected candidates were further confirmed as up-regulated by western blot, and multiple reaction monitoring (MRM) protein quantification. Of these, transforming growth factor beta-induced (TGFBI), latent transforming growth factor beta binding 2 (LTBP2), and asporin (ASPN) were further investigated by immunohistochemistry and found to be overexpressed in a large collection of pancreatic cancer tissues compared to normal and inflammatory tissues. These candidates had not previously been linked to pancreatic cancer at the level of protein expression. TGFBI is a secreted extracellular matrix protein that is overexpressed by several cancer types, and is suggested to promote metastatic progression [50-53]. TGFBI may induce dissociation of VE-cadherin junctions, and eventual breakdown of the endothelial barrier via the integrin alpha-v beta-5 and the Src signal pathway, leading to increased cancer cell extravasation [52]. The other candidate protein, LTBP2, is reported to have a role in cell adhesion, and TGF-beta1 increases expression of LTBP2 at the transcriptional level [54,55]. ASPN is a member of the small leucine-rich proteoglycan (SLRP) family. Little is known regarding the function of ASPN in pancreatic cancer, but it is supposed to have a role in TGF-beta/Smad signaling [56].

In another deep mining proteomics study, Takadate et al. [30] used label-free quantification of pancreatic cancer tissues with poor and better survival outcomes, and non-cancancerous pancreatic ducts. Of these proteins, 170 were selected for MRM protein quantification. Of these, fourteen proteins were found to be overexpressed in pancreatic cancer compared to normal tissue. Furthermore, patients whose tumors expressed the proteins enoyl-CoA hydratase 1 (ECH1), olfactomedin-4 (OLFM4), stomatin-like protein 2 (STML2) and glucose transporter member 1 (GLUT1 or GTR2) had significantly worse survival, suggesting that these proteins may have prognostic value, and may serve as new therapeutic targets. ECH1 is an enzyme that functions in the auxiliary step of the fatty acid beta-oxidation pathway. Upregulation of ECH1 is associated with cancer cell proliferation, increased ratio of cells in S phase to G1 phase, and increased migration capacities, and may have an important role in the development of lymphatic metastasis [57]. OLFM4 is an extracellular matrix glycoprotein that regulates cell adhesion and is also considered to be an antiapoptotic factor that promotes tumor growth [58]. STML2 is a member of the stomatin superfamily. It has been identified as an oncogenic-related protein and found to be overexpressed in several cancers [59-61]. STML2 down-regulation can inhibit cancer cell invasion in an MMP2 dependent manner [60]. Also, the up-regulation of STML2 is involved in activation of the MAPK/ERK pathway [60]. GLUT1 is a member of the facilitative glucose transporter family. The GLUT1 protein is considered to be upregulated in tumor cells, which have enhanced metabolism and increased glucose requirements [62]. An increased GLUT1 expression has also been linked to pancreatic cancer invasiveness and poor prognosis [63,64].

Another study by Paulo et al. [65] used label-free quantification of pancreatic cancer, chronic pancreatitis and normal pancreas tissues. Of these proteins, 21 proteins were identified exclusively in pancreatic cancer specimens including annexin 4A (ANXA4) and fibronectin (FN1). ANXA4 is a member of the annexin family of calcium-dependent phospholipid binding proteins. ANXA4 is suggested to be implicated in proliferation, migration and chemoresistance of cancer cells [66,67]. The other candidate, FN1, is a glycoprotein involved in cell adhesion and migration processes. The presence of both cellular and stromal FN1 and its interaction with integrins is necessary for pancreatic cancer progression, and inhibition of FN1 signaling was previously shown be an effective treatment strategy *in vivo *[68].

Matsubara et al. [69] used label-free quantification of plasma from pancreatic cancer patients and healthy controls. Of the MS peaks, a peptide derived from CXC chemokine ligand 7 (CXCL7) was significantly reduced in pancreatic cancer patients. Reduction of the CXCL7 protein in pancreatic cancer was also observed in a validation cohort. Interestingly they found that the combination of CA 19–9 with CXCL7 significantly improved the AUC value of CA19-9 to 0.961. CXCL7 belongs to the CXC chemokine family and is a proangiogenic and proinflammatory cytokine. The reduction of circulating CXCL7 in patients with pancreatic cancer may have a role in the suppression of angiogenesis, and may reflect the hypoxic nature of pancreatic cancer tissue [69]. Plasma CXCL7 may be degraded by exoproteases such as matrix metalloproteinase-9 (MMP9) that are secreted into the plasma by pancreatic cancer cells [70-72].

### Labeled quantification

The labeling of proteins and peptides with stable heavy isotopes (deuterium, carbon-13, nitrogen-15, and oxygen-18) have been used in clinical and pharmacologic settings for decades, but have recently found a major part exploring the possibilities promised by contemporary proteome research. Both metabolic and post-metabolic labeling, by chemical or enzymatic reaction, can be used to incorporate the labeling isotopes. After fragmentation, peptides or reporter ions are used for quantification of the targeted proteins based on their signal intensities in mass spectrometric analysis [73]. The incorporation of isotope-labeling, makes the labeled peptides become the internal standard of the assay. This procedure provides a unique feature, to identify endogenous analytes in the sample, where even highly complex samples can be screened by this methodology [74]. The advantage of utilizing isotope labeled internal standards is also that these protein sequences do not exist naturally in human samples. The methods based on residue-specific chemical derivatization with labeled reagents offers great flexibility are universally applicable, but may sometimes become too complicated, resulting in unwanted side reactions. The metabolic approaches do not require any manipulation of the proteins but are limited to cells where full control of culturing can be obtained [75]. The quantitative analysis can be “isotopic” or “isobaric” with the main difference that isotopic methods (SILAC. ICAT, and ICPL) quantify peptides at the MS level based on ion intensities of light and heavy isotopes of a peptide. While the isobaric methods (TMT, and iTRAQ) quantify peptides at MS/MS level, based on comparison of measured reporter ion intensities [76].

#### *Metabolic labeling*

Stable isotope labeling by amino acids in cell culture (SILAC) is a metabolic isotope-labeling technique incorporating specific amino acids in vivo into mammalian proteins [77-79]. The cell lines are grown in a media where a standard essential amino acid is exchanged by a non-radioactive, isotopically labeled form and thus incorporated into all proteins synthesized. This step has been shown to not influence the growth of the cells. It takes a certain number of cell doublings for the complete incorporation of the isotopically labeled analog into the proteins studied. Proteins from the experimental and control samples are mixed and mass spectrometric measurements are used for relative quantification. It is a simple, inexpensive and accurate technique applicable to any cell culture system. Chemical labeling and affinity purification steps are not involved in this process, but the downside is that it requires living cells. However, Geiger et al. [80] recently developed a method called Super-SILAC for quantitative proteomics on human tumor tissue.

Grønborg et al. [81] was one of the first research teams to provide protein expression database annotations, using SILAC, to compare the secretomes of the human pancreatic cancer cell line PANC1 and the normal pancreas cell line HPDE. Eleven dysregulated proteins were verified by western blot analysis. Five proteins including CD9, perlecan (HSPG2), apolipoprotein E (APOE), stromal cell derived factor 4 (SDF4), and fibronectin receptor/integrin β1 (ITGB1) were subsequently validated by immunohistochemistry on human pancreatic cancer tissue. CD9 is a cell surface glycoprotein that belongs to the tetraspanin family. It is involved in many cellular processes including cell adhesion, and signal transduction, and also plays a critical role in the suppression of cancer cell motility and metastasis [82]. Low CD9 expression is related to poor prognosis in several cancers including pancreatic cancer [82,83]. HSPG2 is a pericellular proteoglycan that controls cell migration, proliferation, and differentiation [84]. HSPG2 can interact with growth factors and facilitate cancer growth and angiogenesis [84,85]. APOE is a ligand for low density lipoprotein receptors [86]. Besides its role in cholesterol transport and metabolism, APOE is also involved in several diesease processes. APOE can affect cell growth, proliferation, and immune response [87,88]. APOE is elevated in several malignancies including pancreatic cancer [89,90]. It has been suggested tumor promoting effects of APOE is may be related to the inhibition of TNF-alpha [91]. SDFs refer to a group of proteins that are generated by stromal cells. While it has been shown that SDF1 is overexpressed in aggressive pancreatic tumors and high levels of SDF1 are linked to a poor prognosis [92], the significance of SDF4 is in pancreatic cancer is less known. ITGB1 is a membrane receptor in the integrin family, and is involved in cell adhesion and can facilitate metastatic spread of cancer cells through interactions with the extracellular matrix [93].

In an additional recent study, Yu et al. [94] used SILAC to collect secreted proteins from the human pancreatic cancer cell line CAPAN-2. The resulting stable isotope labeled proteome (SILAP) standard was added to each pooled sample from pancreatic cancer and benign pancreatic disease. Proteins were separated by isoelectric focusing before 2D-LC-MS/MS analysis. Independent validation was performed with ELISA for two proteins, intercellular adhesion molecule 1 (ICAM1) and basal cell adhesion molecule (BCAM). ICAM1 has a role in cell–cell and cell–extracellular matrix adhesion. ICAM1 has previously been reported to be overexpressed in pancreatic cancer, and serves as an important docking point for polymorphonuclear cells that functionally promote tumor cell metastasis [95,96]. BCAM is a laminin receptor and a member of the immunoglobulin superfamily. Little is known about the role of BCAM in pancreatic cancer, but laminin alpha 5 is widely expressed in basement membranes, and therefore it has been suggested that BCAM may play a role during the process of tumor invasion [97].

#### *Isotopic labeling*

Isotope-coded affinity tags (ICATs) is a class of reagent, consisting of three functional elements; a chemical group reactive towards cysteines (thiol groups), an isotopically coded linker, and an affinity tag (biotin) used in the isolation of ICAT-labeled peptides. It is a highly accurate technique used on either cells or tissue for relative quantification because it is based on stable isotope dilution. One heavy (deuterium) and one light (hydrogen) form exist for every reagent. The two samples being compared are introduced to the ICAT reagents that covalently attached to each cysteinyl residue in every protein. One sample receives the light and the other receives the heavy form. The two samples are then combined and reacted with proteolytic enzymes to produce peptide fragments. The ICAT-labeled (cysteine-containing) peptides with their biotin tag can then be isolated by an avidin affinity chromatography. The isolated peptides are then separated and analyzed by a liquid chromatography-MS/MS procedure. An automated multistage MS generates both the quantity and sequence identity of the originally tagged proteins. The ratio of signal intensities of peptide pairs between the samples (differing with 8 Dalton mass) utilized with MS provides an accurate measure of the relative quantities. It is thus important to have the same ratios of the original amounts of proteins from the two samples through the whole process [98]. Improvements have been made using other isotopes than deuterium. Most recently an approach using Carbon-13 as the heavy element resulted in an increase of the number of proteins identified per experiment. The retention time shift, a common problem with labeled techniques caused by significantly altered hydrogen bonding of deuterated compounds, could also be avoided with this approach [99].

In a recent study, Chen et al. [100] used ICAT applied to pancreatic cancer tissues and matching normal pancreas samples. Annexin A2 (ANXA2) was identified as a biomarker for pancreatic cancer. ANXA2 is part of the annexin family of proteins. It has been shown that ANXA2 can mediate epithelial to mesenchymal transition, invasion, and metastases in pancreatic cancer, for example through translocation from the cytosol to the cell membrane [101,102]. Chen et al. [100] validated ANXA2 and the other discovered protein, ITGB1, using western blotting, and immunohistochemistry.

In another study, Chen et al. [103] used ICAT of pancreatic juice from a pancreatic cancer patient and normal controls. One of the identified proteins, insulin-like growth factor binding protein-2 (IGFBP2), was further validated by western blotting to be elevated in pancreatic cancer juice and overexpressed in pancreatic cancer tissue. IGFBP2 has been found to be overexpressed in many malignant tissues, including pancreatic cancer [35,104,105]. Insulin-like growth factors (IGFs) and IGF binding proteins (IGFBPs) are responsible for growth of neoplastic cells through autocrine and paracrine mitogenic signals [104].

Complementary to these studies, Pan et al. [106] used stable isotopic heavy and light acrylamide labels of plasma from pancreatic cancer patients, chronic pancreatitis patients and non-pancreatic disease controls. They found that metallopeptidase inhibitor 1 (TIMP1), a natural tissue inhibitor of matrix metalloproteinases (MMPs) which degrade extracellular matrix, was identified as a biomarker candidate for pancreatic cancer. TIMP1 has been previously shown to reduce pancreatic cancer cell growth, metastasis, and angiogenesis, while increasing tumor apoptosis [107]. Pan et al. [106] found that TIMP1 together with ICAM1 demonstrated a significantly better performance than CA19-9 in distinguishing pancreatic cancer from the controls.

### Isobaric labels

Tandem mass tags (TMTs) is a MS/MS-based strategy using isotopomer isobaric labels for accurate quantification of peptides and proteins. The tags are comprised of different regions. The sensitization and mass differentiation group are cleaved during collision-induced dissociation (CID) and constitutes the TMT fragment being detected and used for quantification. Unlike other methods the pairs of TMT-tagged peptides have the same overall mass due to a mass-normalization group, and thus co-migrate in chromatographic separations leading to a more precise quantification. The co-migration means that the MS signal peak for each peptide pair will not be split, improving sensitivity in MS mode. The reactive functionality of the tags can be manipulated, so that it allows a coupling and labeling of any peptide to be made. The second generation tag differ from the first by introducing an additional fragmentation-enhancing group [108]. The TMT labeling allows for simultaneous identification and relative quantification, on MS/MS fragmentation. Improved 6-plex TMTs have been designed to be able to compare up to six different extracts. It uses 13C or 15 N instead of H in the former 2-plex TMTs that guaranties co-elution during the LC separation [109].

An elegant example was recently presented by Sinclair et al. [110] who showed that they could use amino-group labelling of proteins with TMT of sera from pre-diagnosis pancreatic cancer cases, and matched healthy controls samples. Examples of consistent protein expression changes all the way up to clinical diagnosis of pancreatic cancer, were; gelsolin (GSN), apolipoprotein A4 (APOA4), coagulation factor 12 (F12) and lactotransferrin (LTF). GSN is an actin-capping protein and GSN levels are actively downregulated in pancreatic cancer and the ubiquitin-proteasome pathway is an important contributing factor for this effect [111]. APOA4 levels are also significantly decreased in patients with pancreatic cancer [112]. F12 may facilitate cancer cell metastasis by transforming monocytes-macrophages toward tumor-associated macrophage-like cells [113]. LTF plays important role in innate immunity, and it has been shown that LTF is significantly down-regulated in pancreatic cancer [114]. LTF may act as a tumor suppressor by suppressing AKT signaling [115].

Isobaric tags for relative and absolute quantitation (iTRAQ) is based on covalent labeling of the N-termini and lysine side chains. It contains one reporter group, an amine-reactive group, and a balancing group between them. It can either be 4-plex or 8-plex, both isobaric with a mass of 145 to 305 Da, respectively. The balancer group is not detectable by MS because it is liberated as a neutral fragment within the assay. The reporter group is cleaved during CID generating quantifiable isotope fragments. The quantification is exclusively done on MS/MS level, reducing chemical noise and improving precision compared to MS-based techniques [116]. With iTRAQ, up to eight relative quantifications can simultaneous be done in a single run. A disadvantage with isobaric labels is that there may be interference due to overlapping precursor ions. Several approaches have been conducted to overcome this limitation, such as using an additional step with isolation and fragmentation [117,118].

Pan et al. [119] used iTRAQ and ICAT of immediate precursor of pancreatic cancer (PanIN 3), pancreatic cancer, chronic pancreatitis and normal pancreas tissues. Network analysis of the proteins identified c-MYC as an important regulatory protein in PanIN 3 lesions. Furthermore, three of the overexpressed proteins, laminin beta-1 (LAMB1), galectin-1 (LGALS1), and actinin-4 (ACTN4) were validated by immunohistochemistry analysis. All three of these proteins were overexpressed in the stroma or ductal epithelial cells of advanced PanIN lesions as well as in pancreatic cancer tissue. LAMB1 belongs to a group of basement membrane proteins that significantly enhance the invasive behavior of pancreatic cancer cells [120]. GAL1 is a beta-galactoside-binding lectin that can induce chemokine production and proliferation in pancreatic stellate cells which promote pancreatic fibrosis and tumor progression [121,122]. Actinin-4 is an actin-binding protein associated with enhanced cell motility, invasive growth, and poor survival in pancreatic cancer [123,124]. E-cadherin regulates the association between beta-catenin and actinin-4 [125].

## Targeted protein quantification

**Figure 1:**
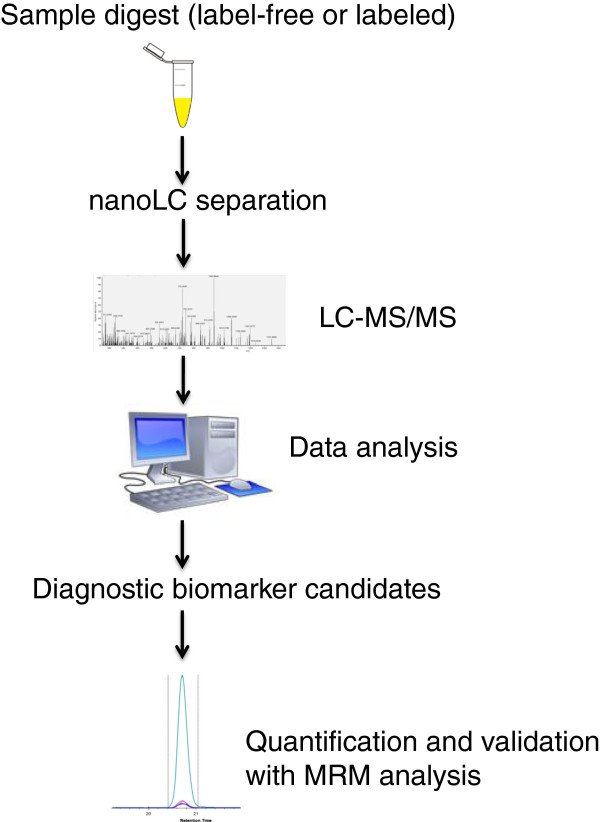
Protein assay development in pancreatic cancer from discovery to validation.

Following discovery-based deep mining studies, several biomarker candidates need to be selected for further studies, based on phenotype specificity and/or direct mechanistic role in the disease. Using more targeted protein analyses such as selected reaction monitoring (SRM; or MRM plural), these biomarkers can then be validated in large patient cohorts (see Figure 1). Targeted protein analyses allow simultaneous quantitation and identity confirmation with high sensitivity in a single LC-MS/MS run. By utilizing isotop labeled internal standards, the absolute abundances of each protein can be determined.

### MRM assay principles

Targeted analysis of single or multiple proteins (multiplex) using LC-MS technology in MRM mode, has gained extensive attention recently. MRM is a mass spectrometric scan type with the highest duty cycle that can monitor one or more specific ion transition(s) at high sensitivity. MRM assays are becoming a true complement to ELISA assays. It has already been shown that an MRM assay is capable of monitoring 50 proteins simultaneously [126]. In order to push sensitivity of low abundant proteins in terms of technology a sample preparation step is needed. To be able to compete with the sensitivity of ELISA, which is capable of quantifying proteins at low picogram per milliliter levels, immunoaffinity-assisted LC-MS/MS analysis has emerged. Recent developments in immunoaffinity-assisted LC-MS/MS analysis have been able to show picogram per milliliter sensitivities [127,128]. MRM is a more cost-effective and timesaving assay than standard ELISA assays and has the capacity to quantify multiple proteins in one analysis with high reproducibility.

The development of MRM assays is based on the selection of one or several target peptides for each protein within the assay. The samples are digested, commonly with trypsin, followed by LC-MS. Chemically stable isotope-labeled peptides are most commonly used for MS-based absolute quantification of proteins in the multiplex MRM assay developments.

### *In Silico* processing

When developing an MRM assay, the first step is the *in silico* processing. *In silico* methodology is used specifically in the selection process, to build the multiplex protein assay [129,130]. The selection of a unique peptide representative for the targeted protein or a specific isoform thereof is a critical step to generate true and replicable results. These peptides are termed proteotypic peptides (PTPs) and from these the fragment ions generating optimal signal intensity and discriminate the targeted peptide have to be identified. To obtain the most reliable selection, the fragment ion masses of the targeted peptide can be calculated and experimentally tested by SRM measurements on a triple quadrupole instrument. For time-saving and help in the selection of target peptide set information can be gathered from previous experiments or from the emerging online databases (including PRIDE, PeptideAtlas and Human Proteinpedia) [131].

To get a more reliable quantification, at least two peptides should be monitored for each targeted protein where any divergence in regulation can be assumed to stem from post-translational modifications and/or overlapping protein sequences [131]. Studies have evaluated the application of MRM in determining post-translational modifications. The technique MRM-initiated detection and sequencing (MIDAS) has been shown to be highly sensitive in detection of protein phosphorylation and to identify sites of acetylation [132,133].

The complexity of mammalian systems makes it possible for multiple proteins to act as precursors of a single peptide and thus result in under- or overestimation of the discovered marker candidates [134]. Peptides containing side chains from methionine or tryptophan residues are prone to oxidation, while peptides containing glutamine or asparagine residues may convert to glutamate or aspartate. Caution should be raised towards peptides, observed in shotgun analyses, with missed cleavages or non-tryptic cleavage sites. Two neighboring basic amino acids at either cleavage site are prone to high rate of missed cleavages. It is important to have a unique set of transitions; otherwise precursor/fragment ion pairs with similar masses can create unspecific signals. A high abundant protein or one generating a strong signal might thus disturb the signal from the protein being studied [131].

Several systems exist to support the procedure. They can help in the selection of targeted protein sets, finding representative PTPs, and manage and validation of transitions to get more reliable data. The dedicated analysis software “Skyline”, supports the direct picking of peptide, precursor and transition *in silico* from proteins and fragment peptides [135]. Other platform-specific tools include TIQAM, MRMPilot, SRM Workflow Software, Verify, and Optimizer [131]. MRMer is another open source software tool, for the analysis of data generated by highly complex MRM-MS experiments. It provides a rapid visual inspection of over 1000 precursor-product pairs [136].

The highest sensitivity of MRM-MS requires optimal peptide fragmentation and maximal transmissions of the desired product ions, which is dependent on optimal settings of parameters that includes collision energy, cone voltage, and de-clustering potential. Methods for fine-tuning the parameters have been developed [137]. Algorithms helping to identify inaccurate transitions by interfering signals or inconsistent recovery among replicate samples are being developed to reduce the need for manual and subjective inspection of data, and thus improve the overall accuracy and minimize errors [138].

### The MRM technology platform of biomarkers

The primary benefits of MRM assays are their multiplexing capabilities. This allows panels of proteins to be quantitated in a single assay cycle. In principle, any protein can become an analyte. Although antibody-based techniques, such as ELISA, represent the current gold standard for protein biomarker quantification, the development of high quality antibody assays requires time and resources and has been a limitation in biomarker translation. The multiplexing capabilities and the generic concept of the MRM assay makes it superior to current ELISA assays [139]. In recent years, several studies have demonstrated the value of using targeted MRM proteomics for candidate pancreatic cancer biomarker validation.

The studies by Tortoi et al. [49] and Takadate et al. [30] used protein deep mining followed by MRM analysis as described in the previous section.

Pan et al. [85] applied an MRM-based targeted proteomics platform to directly detect candidate biomarker proteins in plasma. The study included patients with pancreatic cancer and chronic pancreatitis, as well as healthy individuals. Plasma was first depleted to remove albumin and IgG. Three of the 5 candidate proteins, including GSN, lumican (LUM; a member of the SLRP family), and TIMP1, demonstrated an AUC value above 0.75 in differentiating pancreatic cancer from the controls.

Yoneyama et al. [140] used MRM quantification of proline hydroxylation at residues 530 and 565 of alpha-fibrinogen. The results indicate that PTMs can be used as biomarkers of pancreatic cancer, and also identify CA19-9-negative patients.

## Large meta-data studies

Large scale studies with *meta data* processing is a new development area, where the analysis of archived biobank samples is and will play a major role is within the field of new biomarkers and new diagnostic developments. It can be seen that both industry as well as the academic field invest and are adding large resources, searching for approaches to improve on the discovery successes where new technology plays a central role. Here, the ENCODE initiative has over the years produced an extensive DNA-sequence resource that is being utilized by the Proteomics Community [141]. The human genome sequencing platforms including the latest generation of deep-sequencing platforms, allows us to integrate new data with genetic risk factors, aligning with proteomics data [19,142].

**Figure 2:**
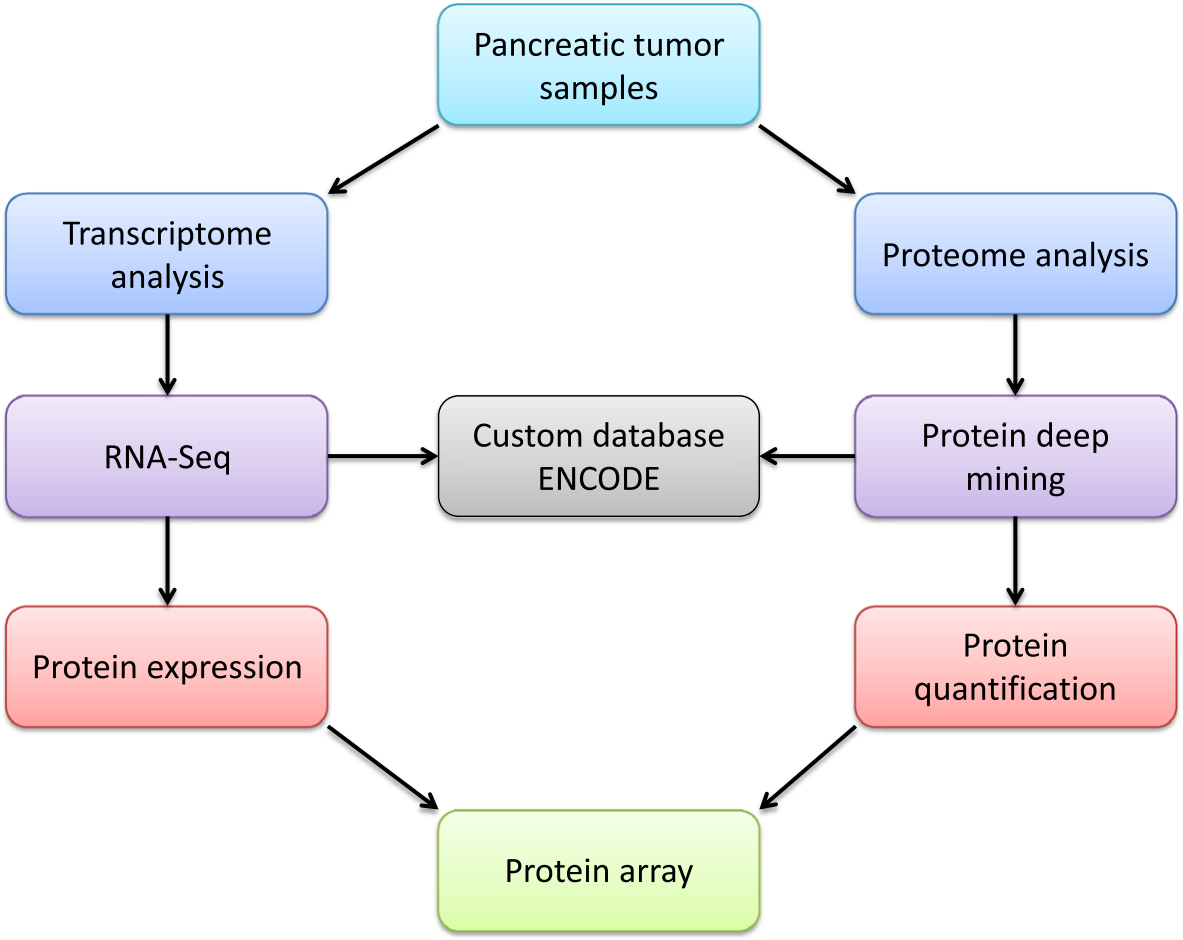
**Workflow for biomarker translation in pancreatic cancer using large-scale *meta data *** **processing.**

To get a comprehensive understanding of protein changes in disease will require quantitative information from proteomic, transcriptomic and genomic data. Recently, whole transcriptome analysis using RNA sequencing revealed an average of 109 million mapped reads in pancreatic cancer and 877 genes and isoforms identified as showing significant expression changes [143]. We are proposing an integration of transcriptomic and proteomic data in order to enhance biomarker translation, as shown in Figure 2. We suggest that pancreatic tumor samples are analyzed with quantitative proteomics and RNA-Seq. Identification and quantification are performed at the protein and transcript levels, and a custom protein database is generated from the RNA-Seq data. Comparisons are made between protein and transcript data, and the custom protein database is used to search for proteins. Validated protein identifications are queried against protein databases such as Human Protein Atlas [144] and neXtProt [145] to determine levels of protein evidence, and quantitative proteomic data are used to generate networks in Ingenuity Pathway Analysis. Proteins and transcripts are related to genomic data in ENCODE.

## Summary

The characterization of the pancreatic cancer proteome will inevitably increase our knowledge of pancreatic tumorigenesis. Many of the hitherto identified proteins are involved in crucial steps in pancreatic cancer progression such as cell proliferation, adhesion, migration, invasion, metastasis, immune response and angiogenesis. These new protein biomarker candidates will provide essential information for the development of screening tests and targeted therapies. Quantitative mass spectrometry may also be useful in identifying the signaling that is aberrantly activated in pancreatic cancer [146] or identifying the metabolites from pancreatic cancer patients [147]. With the introduction of improved proteomic techniques we are thus heading towards a more personalized medical (PM) approach, with the ability to provide useful guidance in selecting the right patient for PM-treatment. The technical development in mass spectrometry instrumentation, sample preparation methods, and protein identification methods is rapidly changing the way clinical studies measure biomarkers in patient samples. We can now measure proteins at low abundance levels and there are a number of studies that have utilized trace enrichment utilizing antibody probes to reach down to picogram per milliliter sensitivities [127,128]. A recent paper by Lopez and colleagues provide data where they have quantified a large number of proteins utilizing immunoaffinity enrichment [148]. In addition to the measurement advancements, commercial dedicated small robotics is commonly used to handle the multiple step sample preparations, and as such become a necessary and efficient step in the work process within clinical studies. It can be foreseen that sequence based mass spectrometry assays and platforms that provide multiplex read-out will gain much attention, and that it will become a milestone in future clinical science. The ability to screen patient samples with panels of hundreds of protein biomarkers with absolute quantitation provides an increased value. The future generation of personalized medicine where the individual patient will receive the best possible medicine for the correct disease will drive the entire diagnostics field forward. Studies such as the NCI Clinical Proteomic Technology Assessment for Cancer initiative (NCI-CPTAC) that are publicly available, are examples of milestone achievements that are changing the everyday routines in clinical sciences. The outline details and information on the initiative of the national cancer institute can be found at http://proteomics.cancer.gov/programs/cptacnetwork. The new initiative provides data on applying new technologies for biomarker verification in plasma [149].

## Concluding remarks

In respect to perform deep mining protein sequencing on pancreatic cancer patients by mass spectrometry, the Chromosome-Centric Human Proteome Project (C-HPP, http://www.c-hpp.org) provides resources that enables novel annotations to be verified. The C-HPP consortia aimes at identifying all proteins encoded by the human chromosomes [19]. The link to the ENCODE Consortium, has recently been presented where genomics exon verifications can be identified on a protein sequence level [150].

The challenge is to couple the gene sequences to relevant amino acid sequences utilizing the ENCODE bioinformatic engines. This approach was recently presented in a recent brain tumor study, that can be adapted to pancreatic cancer [151].

## Competing interests

The authors declare that they have no competing interests.

## Authors’ contributions

DA and LA conducted the literature search and drafted the manuscript. AS, CW, MR, GMV, and RA revised the manuscript. GMV and RA conceived the study. All authors reviewed and approved the final manuscript.
